# Progress in Sensors for Monitoring Reinforcement Corrosion in Reinforced Concrete Structures—A Review

**DOI:** 10.3390/s22093421

**Published:** 2022-04-29

**Authors:** Dmitry Shevtsov, Nhat Linh Cao, Van Chi Nguyen, Quoc Quang Nong, Hong Quan Le, Duc Anh Nguyen, Ilya Zartsyn, Oleg Kozaderov

**Affiliations:** 1 Faculty of Chemistry, Voronezh State University, Universitetskaya pl. 1, Voronezh 394018, Russia; zar-vrn@mail.ru (I.Z.); kozaderov@vsu.ru (O.K.); 2 Coastal Branch, Vietnam-Russia Tropical Centre, Nguyen Thien Thuat St., 30, Nha Trang 57127, Khanh Hoa, Vietnam; nguyenvanchirvtc@gmail.com (V.C.N.); nquocquang5@gmail.com (Q.Q.N.); quanttndvn@gmail.com (H.Q.L.); nda.ttndvn@gmail.com (D.A.N.)

**Keywords:** corrosion of reinforcement, reinforced concrete, non-destructive monitoring, smart constructions

## Abstract

Non-destructive monitoring methods and continuous monitoring systems based on them are crucial elements of modern systems for the management and maintenance of assets which include reinforced concrete structures. The purpose of our study was to summarise the data on the most common sensors and systems for the non-destructive monitoring of reinforced concrete structures developed over the past 20 years. We considered systems based on electrochemical (potentiometry, methods related to polarisation) and physical (electromagnetic and ultrasonic waves, piezoelectric effect, thermography) examination methods. Special focus is devoted to the existing sensors and the results obtained using these sensors, as well as the advantages and disadvantages of their setups or other equipment used. The review considers earlier approaches and available commercial products, as well as relatively new sensors which are currently being tested.

## 1. Introduction

Reinforced concrete (RC) structures serve as the basic composite material for modern civilisation. This composite material is used to construct industrial buildings, energy and transportation infrastructure, and social facilities. The unique properties of RC make it possible to implement any technical and architectural solution and construct buildings of virtually any size, form, and function.

RC is a durable material with an expected repair-free service life of up to 100 years, according to EN 1991 (2002–2006 Eurocode 1: Actions on structures). However, there have been numerous cases, when such structures required maintenance long before the end of the design service life. Without timely and proper maintenance, RC structures are prone to collapse. The most common cause of premature destruction of RC structures is the corrosion of reinforcements [1]. Corrosion is most often induced by chlorides being the components of salt water and antifreeze reagents, as well as products of the chemical industry [2]. Carbonation of concrete is also a common cause of corrosion of steel reinforcement in concrete [3]. The destruction of RC structures proceeds in several stages, the most common being the following [4]: loss of passivity, the cracking and flaking of the protective layer accompanied by impaired adhesion between the reinforcements and the concrete, and finally, collapse. At the moment, countries with developed market economies spend up to 3–5% GPD to mitigate the consequences of the corrosion of steel reinforcement bars [5]. One of the main tasks facing studies of metal corrosion in the 21st century is to reduce these costs.

The corrosion of steel reinforcement bars can be minimized by adjusting the composition of concrete (selecting the type of cement, additives, and corrosion inhibitors) and proper construction works. These measures provide for the primary protection of RC during the manufacturing stage. The secondary protection (polymer and cement coatings, hydrophobisators, migrating corrosion inhibitors) is applied after manufacturing and during operation in corrosive environments.

The cost of the service life of any structure comprises the costs required during all stages, from construction to disposal. It can be reduced by selecting technological solutions that would ensure optimal construction and operational costs [6]. In order to do this, it is necessary to employ methods for assessing the effectiveness of the primary and secondary protection, and forecasting the repair-free service life with regard to the level of corrosivity of the environment and the condition of the RC. Non-destructive monitoring methods (and continuous monitoring systems based on them) are a crucial element of the systems for the management and maintenance of assets which include RC structures.

Corrosion of reinforcement steel in concrete proceeds as an electrochemical process [7]. Therefore the most common methods used to identify the state of reinforcement (passivity or corrosion) or the corrosion rate are electrochemical methods. Physical methods are also growing in popularity, since they can be used to assess the development of the corrosion process based on indirect parameters such as permeability change, reduction of the level of adhesion at the steel/concrete boundary, and cracking caused by the accumulation of corrosion products.

Currently, there are a significant number of reviews on the topic of sensors for assessing the corrosion condition of reinforcement in concrete. Some of them are aimed at a detailed discussion of specific methods: electrochemical [8], control of chloride content [9], fiber-optic [10], piezoelectric [11], etc. Other reviews cover groups of methods and discuss in detail the basics of methods, as well as give some specific applications of sensor designs, most often in laboratory conditions [12]. At the same time, researchers often play insufficient attention to systems that are used on real structures and ready-made commercial solutions.

The purpose of our research was to review the existing literature on the most common sensors and non-destructive monitoring systems for RC structures. Of particular interest are sensors which are used or can be used for remote data collection and transfer systems, i.e., systems that do not require the constant presence of engineers close to the examined structures. The article focuses on specific sensors based on the existing principles. The scope of our study does not include a detailed discussion of the physicochemical principles of particular methods. The article covers a period of the past 20 years and reviews earlier approaches and available commercial products, as well as relatively new sensors during various stages of testing.

The review consists of the following sections:

2. Electrochemical Methods.

○ 2.1. Half-Cell Potential (HCP) Sensors.
○ 2.2. Concrete Resistivity (CR) Measurement Sensors.
○ 2.3. Macro- and Microcell Sensors.
○ 2.4. Linear Polarisation Resistance (LPR) Sensors.
○ 2.5. Galvanostatic Pulse Technique (GPT) Sensors.
○ 2.6. Electrochemical Impedance Spectroscopy (EIS) Sensors.
○ 2.7. Chloride Monitoring Sensors.
○ 2.8. pH-Sensors.

3. Physical Methods.

○ 3.1. Fibre Optic Sensors (FOS).
  ▪ 3.1.1. Fibre Bragg Grating (FBG) Strain Sensors.
  ▪ 3.1.2. Long Period Fibre Grating (LPFG) Refractive Index Sensors.
  ▪ 3.1.3. Brillouin Optical Time Domain Reflectometry Sensors.
○ 3.2. Elastic Wave Sensors.
  ▪ Piezoelectric sensors.
○ 3.3. Hall Effect Sensors in an Electromagnetic Field.

4. Integrated Sensor Systems.

## 2. Electrochemical Methods

Electrochemical assessment methods allow identifying the corrosion of steel reinforcement bars in concrete either directly or indirectly, based on the changes in the properties of the concrete cover. The principles underlying these methods are based on the quantitative relations between the assessed parameters such as, for instance, the relation between the circuit voltage and the concentration or activity of particular chemical elements, or the presence of ions in the corrosive environment, etc. [13]. Described below are the most common electrochemical methods for monitoring the health of reinforcements and sensor systems based on them, including commercially available ones. The methods are listed from the most to the least popular.

### 2.1. Half-Cell Potential (HCP) Sensors

Measuring the free corrosion potential of steel reinforcementsts (*E*_cor_) on the surface of concrete is one of the earliest methods for assessing the corrosion condition of RC. The first articles on the topic were published in the 1970s [14,15].

On the whole, half-cell potential measurements present a reliable qualitative method, which has been proved by a number of laboratory [16,17] and field [18,19,20] studies. This method has been adopted as standard in a number of countries [21,22,23] and is widely used. The generally accepted values of *E*_cor_ and the corresponding corrosion conditions of reinforcement are given in Table 1. The drawbacks of the HCP method include the lack of a fixed range for the measured potential, the dependence of the results on the temperature and the level of moisture in concrete, and the effect of the films of the coatings and hydrophobisators on the concrete surface.

At the moment, portable sensors are the most popular. They consist of a voltmeter with high input impedance and a reference electrode providing for the consistency of the measurements performed during in situ studies. The most common reference electrodes are copper/copper sulphate and calomel electrodes. The devices have different commercial names in different countries: Canin+ or Profometer Corrosion produced by Proceq, Switzerland; Elcometer 331T by Elcometer, the UK; Giatech iCOR by Giatec Scientific Inc., Ottawa, ON, Canada; Armkor-1 by InterPribor, Russia, etc. The devices differ in their functions, which range from simply measuring and displaying the circuit voltage to mapping the potentials and determining the areas most prone to corrosion on site (the data are not processed by a computer). The application of such devices requires engineers to be in proximity to the examined structures [19].

Of more convenience for remote continuous monitoring systems are sensors, which can be embedded into concrete in the areas most prone to corrosion [24]. There are studies describing sensors based on copper/copper sulphate and silver chloride [25] electrodes that were embedded into concrete. However, the problem of maintaining the stability of such reference electrodes when used with liquid electrolyte solutions have not been solved yet; stability can be lost and some elements can even be destroyed by the alkaline medium of concrete, resulting in the contamination of the concrete with the components of the solution.

Jin et al. [26] suggested using a solid MnO_2_-based reference electrode, which allows for polarisation measurements by means of the HCP method, linear polarisation, and electrochemical impedance spectroscopy. Muralidharan et al. [27] confirmed the effectiveness of MnO_2_-based electrodes for concrete embedded sensors. Later, Karthick et al. [28] suggested a modified reference electrode based on graphene oxide-manganese oxide (GO-MnO_2_), which demonstrated the ability to function stably for at least two years in concrete.

Chand et al. [29] suggested a new method of HCP measurement by means of two coils functioning according to Faraday’s law of electromagnetic induction. Although this approach can hardly become widespread, it demonstrates that researchers today have a wide range of instruments to solve the problem.

We should note that there are hardly any sensors that only monitor the free corrosion potential. Most commonly, integrated systems are used which monitor several parameters simultaneously (pH, chloride concentration, microcell current, etc.) or systems with reference electrodes for polarisation methods. In the latter case, the free corrosion potential is an additional parameter. Taking into account the qualitative nature of the method and the presence of undefined values, this approach is quite reasonable. Integrated systems are discussed in a separate paragraph at the end of this review.

### 2.2. Concrete Resistivity (CR) Measurement Sensors

Measuring the electrical resistivity of concrete is another popular method for monitoring the corrosion condition of reinforcement bars [30,31,32]. There is a linear dependence between the electrical resistivity of concrete, moisture content, and the concentration of soluble salts (including chlorides) in concrete [33]. It is known that under otherwise equal conditions, low resistivity is related to rapid electrochemical processes. However, the dependence of CR on a number of factors, including temperature, relative humidity, amount of atmospheric precipitation, etc., significantly impairs the interpretation of the results obtained during the monitoring of resistivity [34]. Therefore, it is only possible to estimate the probability of corrosion. The criteria are given in Table 2.

Nevertheless, the CR measurement technique is widely used [35] and is traditionally considered complementary to the HCP technique [30,36]. There was a long-term study (over 5 years long) on a specimen of cracked concrete performed in Rødbyhavn (Denmark) [37]. The concrete was subjected to spraying and immersed into water and studied using the HCP and CR techniques based on multicoil electrodes with additional temperature control.

The Wenner probe is becoming more popular for CR measurements performed on site [38]. The probe consists of four metal electrodes arranged on the same line at a specific distance from each other. An electric current (alternating or direct) is applied to the first and the last electrode, and the difference in the potentials is measured for the other electrodes. Thus, a dependence between the current values and the difference in potentials is obtained for several pairs, and the resistivity of the concrete is determined based on the cell constant.

**Figure 1:**
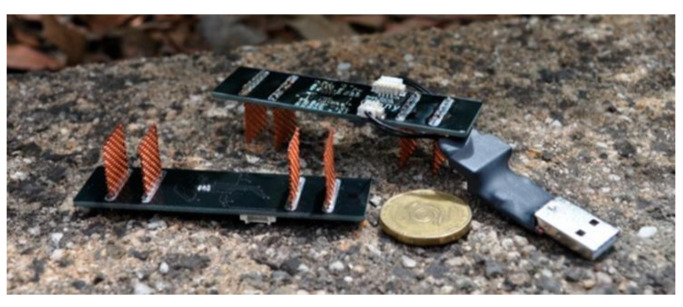
Examples of sensors for determining CR. A four-electrode sensor with USB connection: the external electrodes are designed to generate electric current, and the internal ones are to register the potential difference [41].

There are also in situ sensors embeddable in concrete. Thus, Priou et al. [39] used a multi-electrode sensor in combination with a ROTRONIC humidity sensor and a Pt100 temperature sensor to monitor the corrosion process in an RC wharf. The authors used an ABEM terrameter LS, designed for geophysical studies [40], to collect data under direct current for 18 months. The study was designed to provide detailed information on the rate of penetration of chlorides into concrete in various regions of the wharf without taking the core samples and violating the integrity of the structure. It also helped to test the method for assessing the effect of the distance between reinforcement bars on the results of the measurements of electrical resistivity of concrete. Corva et al. [41] detailed the functioning of a four-electrode probe on a breadboard with USB connection performing measurements under direct current (Figure 1). The USB interface allowed for data transfer to any PC and thus removed the need to design special means of data transfer.

Halabe et al. [42] used sensors comprised of two plain carbon steel plates embedded within concrete cubes. The concrete cubes were tested in a laboratory environment and on site (rehabilitated bridge columns). The study suggested using resistivity sensors in conjunction with commercially available temperature and humidity sensors for a more accurate assessment of the potential for corrosion. A similar device based on two metal rods was suggested by Chi et al. [43]. Simultaneous control of the listed parameters made it possible to increase the accuracy of forecasting the corrosion condition of the reinforcement.

Kamat et al. [44] used embeddable multi-ring sensors with stainless steel electrodes. Their construction made it possible to measure the CR at different depths simultaneously, both in laboratory conditions and on site. As a result of their study, the authors elaborated the dependences of the chloride penetration rates obtained earlier.

The above described monitoring techniques based on HCP and CR measurements are of a qualitative nature and cannot be applied to estimate the rate of destruction of reinforcements in concrete. It is probably for this reason that the sensors based on these techniques are not often used in systems for the continuous monitoring of the condition of steel reinforcements in concrete structures. When installing HCP and CR sensors on existing structures, the detection of fittings by non-destructive methods is required beforehand. One of these is the pacometric test, which is described in detail by Biondi and Frunzio [45].

However, the simplicity of the required equipment and the possibility to examine large regions over short time intervals make these techniques an important tool that can be used for the non-destructive identification of regions with high potential for the corrosion of steel reinforcements in concrete.

### 2.3. Macro- and Microcell Sensors

Sensors based on galvanic macro- and microcells are widely used to estimate the intensity of steel reinforcement corrosion and the depth of corrosion from the concrete surface. Corrosion intensity is defined as a value which cannot be used to directly estimate the rate at which the metal deteriorates as compared to the degradation due to the gravimetric factor, but can be used to estimate how much faster the corrosion process is. After the calibration with regard to control samples, it is possible to recalculate the results by applying the proportionality constant and obtain approximate values for the corrosion rate of reinforcement [4].

Sensors based on microcells are comprised of metal bars with a length of a few centimeters. Anodes are made of low-carbon steel with a composition similar to that of the reinforcement steel. Cathodes can be made of stainless steel [46,47], titanium [48], copper [46,48], etc. One cathode is placed in close proximity to several anodes. There can also be additional reference electrodes for the monitoring of HCP (see Section 2.1) of certain half cells.

**Figure 2:**
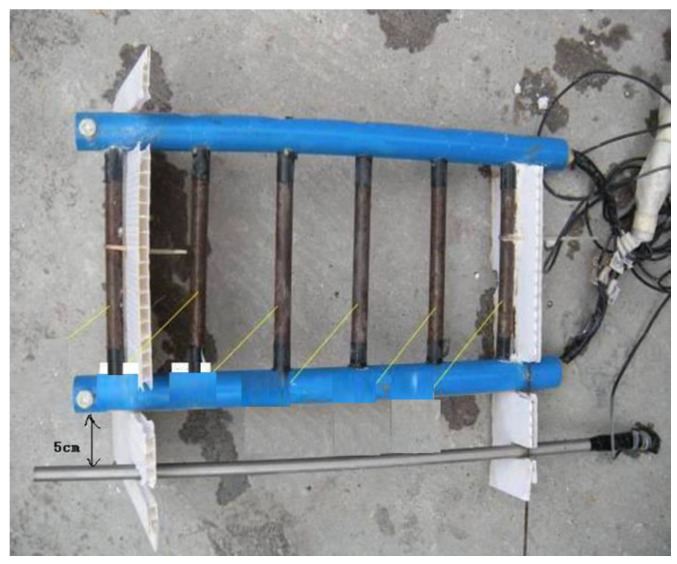
Examples of sensors based on macrocells in the “ladder” type: the inner electrodes are made of reinforcing steel, the outer one is made of stainless steel [52].

The earliest and most popular version of such sensors is the so-called anode-ladder system [49,50,51,52]. The steel bars are arranged in a “ladder” with regard to the reference electrode and can be used to estimate the depth of corrosion in concrete (Figure 2). Such devices also monitor the location of the corrosion front by the difference in the free corrosion potentials between each bar.

Another interesting option is presented by sensors based on macrocells in the form of multi-ring sensors [53]. The sensors are comprised of small ring electrodes isolated from each other and a measurement circuit. The ring electrodes are made of low-carbon steel. When placed in proximity to a cathode made of a more noble metal, the sensor can measure the current in the galvanic cell, and estimate the spread of corrosion spots and their depth depending on the distance from the concrete’s surface.

Valdés et al. [54] studied macrocells based on reinforcing steel and copper with a ratio of areas being 1:1 and also estimated the depth of corrosion. The sensor was comprised of a metal plate (base) and several steel rods placed at various distances from the base. The authors also calibrated the sensor and determined the ranges of values of the current in the galvanic cell corresponding to the passive state and active corrosion of reinforcements based on HCP measurements.

Pereira et al. [55] suggested a sensor for measuring the galvanic current based on a pair of steel/stainless steel reinforcing bars. The sensor included only one galvanic cell and could not be used to estimate the spread of the corrosion front along the concrete cover. However, the results of the experiments with a stainless steel electrode demonstrated that the metal can be used as a cathode. Replacing copper with stainless steel may be a promising solution, since in this case, the concrete does not become contaminated with copper ions, as copper is susceptible to corrosion in chloride environments. On the other hand, the values of galvanic current for a pair of copper/steel electrodes are higher, which makes the sensors based on it more sensitive.

Thus, at the moment, there is no common solution for the construction of sensors based on macrocells and the most effective metal pairs. We should assume that the sensors which can be used to estimate the probability of corrosion at various depths of the concrete cover are more universal, and therefore, more promising.

**Figure 3:**
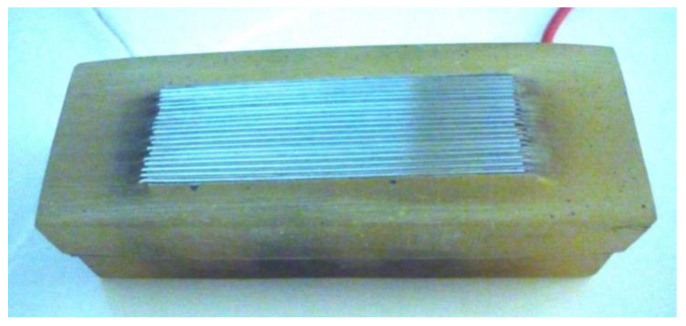
Examples of sensors based on microcells in a bimetallic batch sensor: plates of mild steel and copper with a thickness of 0.1–0.25 mm are separated by a layer of mica with a thickness of 0.1–0.2 mm [56].

Sensors based on microgalvanic cells are comprised of plates of different metals with a thickness of several millimetres. They come in the form of packages of alternating cathodes and anodes (Figure 3) [56]. Such sensors can also be made of single-composition metal plates, with the difference in their potentials being artificially maintained at 20–100 mV [3].

Qiao et al. [57] used model alkali solutions to test microcell sensors based on Mg/graphite and Zn/graphite. Their advantage is that they can generate electric current which serves both as the measurement signal and as a source of power required to transmit this signal over a wireless network. It is assumed that such a system can make the monitoring process much easier, since it does not require numerous meters of connection wires to be embedded into concrete structures to transfer the signal from the recorder to the sensors.

The above described sensors based on macro- and microcells are installed on bridges, in tunnels, and other infrastructure and industrial facilities operating in adverse environments. The sensors are also used to control the quality of maintenance and repair [58], as well as to control the application of corrosion inhibitors, including migrating corrosion inhibitors [59]. The sensors can be embedded either in new or rehabilitated RC constructions. In the former case, the sensors are fixed on the reinforcing bars, after which the form is filled with concrete as usual. In the latter case, cores are drilled in the concrete. The sensors are put in the cores, which are then sealed with a special repair composition. The first method is more preferable with regard to the accuracy of the results, since the concrete cover is homogeneous and has the same properties.

The described sensors were initially designed to be used in remote monitoring systems for a number of reasons. They are simple, inexpensive, and quite compact, and often generate signals without additional polarisation. State-of-the-art technologies can be used to arrange for either the wired or wireless collection, recording, storage, and transmission of data at a relatively low cost. Up to now, there have been a number of various-scale studies, both laboratory [60] and in situ [55], which tested the systems of data collection, storage, and transmission. Thus, installing such sensors in regions susceptible to corrosion and wiring them into a single network enables building management organisations to analyse the condition of the structures as well as plan maintenance and repair works.

### 2.4. Linear Polarisation Resistance (LPR) Sensors

LPR method is based on the fact that there is an inverse relationship between the corrosion current density (*i*_cor_) and the polarisation resistance of the electrochemical reaction. The method has been used to estimate the rate of metal corrosion since the middle of the 20th century [61]. Knowing how *i*_cor_ changes over time, we can estimate the weight loss of the metal or the cross-section loss (Δ*l*) according to Faraday’s law. The most common variant of this method used for the system “steel in concrete” is the LPR method which has been widely used both in laboratories and in situ studies since the 1970s [62].

The measurements are performed with a three-electrode cell (the working electrode is a reinforcement bar, the auxiliary electrode is usually stainless steel, and the reference electrode is the same as used in the HCP technique described in Section 2.1).

The substantiation of the method can be found in a large number of studies [62,63,64,65]. These determined the range of values of the corrosion rate which characterise the danger of utilizing RC structures (Table 3).

LPR takes significantly more time than HCP and CR. Therefore, to optimise the time costs, all the three methods can be used together. Qualitative HCP and CR measurements help to determine the regions with a high probability of corrosion. Then the LPR method is used to determine the rate of deterioration of steel reinforcements.

There are a number of commercially available devices for in situ measurements of the corrosion rate, such as the Gecor 8 by James Instruments; Giatec iCOR by Giatec Scientific, etc. These devices require the presence of a corrosion engineer during the measurements.

Pereira et al. [55] suggested using an embeddable electrode-based sensor together with a commercially available GEOCOR 06 device to estimate the rate of corrosion. Activated titanium was used as a reference electrode. The corrosion rate was estimated based on control samples rather than on the reinforcements of the structure. This means that the composition of the metal used with the sensor must be the same as the composition of the metal in the examined structure.

Jin et al. [25] and Karthick et al. [28] suggested using embeddable LPR-sensors together with HCP measurements to estimate the corrosion rate.

Brown et al. [66] suggested a sensor on a flexible substrate cable. The sensor itself is a standard three-electrode cell. The electrodes were made of corrosion-resistant materials, for instance, gold plated copper.

It should be noted that the LPR method involves a measurement error (the estimated corrosion rate differs from the actual one by 2–4 times) resulting from a simplified calculation procedure [67]. The difference in the rate of uniform and localised corrosion (up to a factor of 10), the effect of the electrical resistivity of concrete, and a series of other factors impair the analysis of the obtained results [68]. However, the easiness and the speed of recording of the polarisation curve make the LRP method a powerful tool for the estimation of the rate of corrosion of steel reinforcements in concrete.

### 2.5. Galvanostatic Pulse Technique (GPT) Sensors

The GPT involves recording the changes in the electrode potential over time, when applying a low galvanostatic pulse (below 50 µA) [69] or after switching off the pulse [70]. The analysis of the obtained potential transient is based on the assumption that the mechanism of the electrochemical reaction on steel reinforcement bars is described by a simple Randles equivalent circuit. The change in the potential can be described by the following expression:

(1) $$E\left(t\right){=I}_{\mathrm{imp}}\cdot \left[{1\;-\;e}^{\frac{{-t}_{1/2}}{R_{p}\cdot C_{\mathrm{dl}}}}\right]{+R}_{\Omega }$$

where E(t) is the measured potential, *I*_imp_ is the impulse current, *t*_½_ is the transition time, *R*_p_ is the polarisation resistance of the electrochemical reaction, *C*_dl_ is the double-layer capacitance, and *R*_Ω_ is the resistance of the medium. Determining the transition time, we can assess the corrosion condition of steel reinforcement bars in concrete (passive or active corrosion). In study [71], the following criteria are suggested: *t*_½_ > 40 s–passive state, *t*_½_ < 25 s–corrosion.

GPT has been used to assess the corrosion of steel reinforcement bars in concrete since the late 1980s [72,73]. At the moment, there are commercially available systems based on the Gecor 8 system and the GalvaPulse sensor with a guard ring as those described in [74,75]. The device is used to determine the corrosion parameters for equation 1. The measurements are performed on site by a corrosion engineer. We did not manage to find any information on remote measurements performed using GTP. However, there are data showing that GPT is more stable and accurate than LPR measurements under adverse conditions, when there is no information about the examined region. It is also more stable and accurate than HCP, LPR, and EIS in the absence of a stable reference electrode [76].

### 2.6. Electrochemical Impedance Spectroscopy (EIS) Sensors

Similar to GTP, the EIS method has been used to monitor the condition of RC since the 1980s [77]. The range of parameters that can be determined using this method is quite wide and includes the electrical resistivity of concrete, the reaction mechanism, the polarisation resistance of the charge transfer reaction, the double-layer capacitance, etc. Lately, there have appeared sensor systems that can register EIS spectra without direct contact with steel reinforcement [78]. However, the inhomogeneity of concrete can cause noise and hinders the analysis of the results of EIS [79].

As we said earlier, Jin et al. [26] suggested a three-electrode sensor for registering the impedance spectra in concrete. Ahmadi et al. [80] obtained the impedance spectra using piezoelectric sensors without polarisation of the reinforcing bars. Sensor plates were installed on rebars embedded in concrete. The authors demonstrated that the suggested devices can be used to determine the time when corrosion starts, and the direction of the corrosion spread, as well as to calculate corrosion-induced weight loss more accurately than can be done by calculating the electrical charge according to the Faraday method. At the same time, the EIS method requires more complex equipment as compared to the LPR method, which makes its in situ application rather problematic.

### 2.7. Chloride Monitoring Sensors

Sensors for chloride concentration or pH level can help to monitor the corrosion condition of RC prior to the beginning of the destruction process [81]. Although there are different views regarding the critical concentration of chloride ions which causes the corrosion of steel reinforcements [82,83,84], all the authors agree that there is such a critical concentration. Thus, monitoring the rate of penetration of chlorides through the concrete cover and determining their concentration on the reinforcement’s surface can be an important part of a comprehensive monitoring system.

There are standard destructive methods used to monitor the concentration of chlorides in concrete. These methods involve core sampling followed by the analysis of aqueous extracts obtained from ground concrete either by titration or by means of the potentiometric method [85,86]. Such methods are not applicable for continuous monitoring because they destroy the concrete cover. Non-destructive chloride sensors can be divided into three major groups according to their operating principle: measuring the electric resistivity of concrete, chloride-selective electrodes, and fibre optic sensors. The first type was, to some extent, discussed in Section 2.2. Below, we will detail the other two types.

Initially, the sensors embeddable into concrete were chloride-selective electrodes, for instance, silver/silver chloride electrodes [25,87,88,89]. Ion-selective electrodes are chemically stable in aggressive environments and easy to manufacture. However, there are a number of factors that can cause inaccurate measurements: changes in temperature and pH and the presence of the electric field. Some sensors deteriorate really fast due to the loss of the electrolyte solution [90].

Im et al. [91] suggested a sensor based on thin iron plates (1.5 mm), fixed in parallel 1 mm from each other on a polyethylene terephthalate substrate. The iron was coated with an anion-exchange membrane sensitive to chlorides. The study demonstrated that the concentration of chlorides can be determined up to ≈1.2 M. Despite the sensitivity and good calibration of the sensor, it has low mechanical resistance and degrades quickly when the concentration of chlorides is high. Therefore, such sensors cannot be embedded into concrete or mortar. The purpose of further studies will be to enhance the durability of such sensors.

Leung et al. [92] developed fibre optic sensors based on a Nufern 780-HP fibre coated with an iron film with the thickness of 25 to 350 nm in order to measure the chloride concentration and determine the threshold value. The readings of the sensor were in good agreement with the results obtained using the galvanic method and macrocells. Unfortunately, the authors did not provide any information about the calibration of the sensors with regard to the actual concentration of free chlorides determined by any well-known method (titration or potentiometric method). Therefore, the study does not provide information on the sensitivity thresholds of the sensors. Laferrière et al. [93] described a sensor based on an indicator dye lucigenin, which is a blue-green, fluorescent chloride-sensitive ion indicator. The study demonstrated the possibility of accurately detecting chlorides within the concentration range from 0.030 to 0.35 M.

### 2.8. pH Sensors

For Ordinary Portland Cement, pH ranges from 12.45 to 13.5 (at 20 °C), and a decrease in pH is expected in aged concrete due to alkali leaching, carbonation, and sulphate attacks for example [94]. A decrease in the pH of concrete resulting from carbonisation (a reaction between carbon dioxide and calcium hydroxide, a component of the cement brick), can lead to the development of the uniform corrosion of steel reinforcement [6]. When localised corrosion is induced by chlorides, there is a critical ratio between the concentrations of chloride and hydroxide ions [95,96]. Therefore, controlling the pH of concrete is important for the comprehensive monitoring of the condition of steel reinforcements.

In their review, Behnood et al. [97] detailed various ways to control the pH of concrete, classified them, and considered the advantages and disadvantages of each method. Therefore, below, we are going to consider some of the well-known sensor designs: ion-sensitive field-effect transistor, fibre optic, hydrogel film, and solid-state pH sensors.

Elements based on metal oxides, including iridium, platinum, palladium, rhodium, titanium, tin, aluminium, and rhenium oxides, are often used as ion-selective electrodes which provide for stable functioning of sensors in concrete. Huang et al. [98] suggested a flexible pH sensor based on iridium oxide. Some electrodes are prepared by means of deposition [99], electrochemical deposition [100], and oxidation [101].

Despite their high mechanical resistance, the effect of the overall ion strength on the readings of such electrodes is to be studied and discussed.

Korostynska et al. suggested using fibre optic sensors for measuring the depth of the carbonisation of concrete [102]. Khalil et al. [103] investigated the use of mesotetraarylporpholactone as a chromophore and demonstrated that it can be used in the pH range of 11.5–13.2. McPolin et al. [104] suggested using a sol-gel based on cresol red with the pH of 8–13.

The main limitation to the use of fibre optic sensors in concrete is the small range of pH values, often below 12, and, for some electrodes, the destruction of the chromophore [97].

Until recently, only the above described electrochemical methods were used to monitor the corrosion condition of steel reinforcement bars in concrete, in particular HCP, CR, and LPR methods. Therefore, they are documented by various standards and regulations, and there is a large assortment of commercially available devices based on these methods. However, at the moment, there are no regulatory documents that would describe the design and assessment criteria for the rest of the above considered electrochemical methods for the continuous non-destructive monitoring of RC and sensors based on them. On the one hand, this creates opportunities for new inventions. At the same time, it inhibits the development of new commercially available solutions. The next stage of research in this field should be the unification and standardisation of the existing approaches.

## 3. Physical Methods

Physical methods of monitoring the corrosion condition of reinforcement bars have become the focus of research only recently (in the past 10–15 years), and many devices are still presented as laboratory samples or prototypes. Below, we consider the most common physical methods and sensors based on them.

### 3.1. Fibre Optic Sensors (FOS)

FOS register changes in the properties of light (photons) transmitted through glass or organic fibres. Depending on the temperature or fibre deformations, there can occur changes in the wavelengths, the energy flux density, frequency, polarisation, or phase. Therefore, it is possible to assess the deformations occurring within concrete as a result of accumulation of corrosion products on the boundary steel reinforcement/concrete. As a rule, FOS is fixed to the reinforcement or in the immediate vicinity so that it is possible to track deformations at the steel/concrete boundary [105]. FOS are very promising for monitoring the corrosion of reinforcement bars in concrete due to their chemical and corrosion resistance, robustness to noise from external sources of electromagnetic radiation, accuracy, and simplicity [106]. The use of carbon nanotubes and the associated shielding of electromagnetic radiation can increase the sensitivity of some sensors [107,108].

At the moment, the most common FOS techniques are Fibre Bragg Grating (FBG) Strain Sensors, Long Period Fibre Grating (LPFG) Refractive Index Sensors, Brillouin Optical Time Domain Reflectometry Sensors, and Shape Memory Alloys (SMA) Sensors.

#### 3.1.1. Fibre Bragg Grating (FBG) Strain Sensors

FBG sensors are sensitive to changes in the reflection values and the lattice period along the optical fibre axis. Monitoring the changes in the reflection signal coming from the lattice makes it possible to estimate the increase in the amount of corrosion products. The application of FBG sensors for monitoring the corrosion of reinforcement bars in concrete has been actively discussed since the 2010s. The technique appears to be quite promising. Currently, most studies focus on the fibre materials and the ways of deploying the sensors in concrete samples. Thus, Mao et al. [109] suggested using Bragg grating fibre covered by epoxy resin for protection. The sensor proved to be mechanically resistant and capable of identifying cracks. However, the authors did not provide any calibration data regarding the relation between concrete deformation and the wavelength. Hu et al. [110] used sensors with double-layer coating (the inner layer was based on silver and the outer layer was based on Fe-C). The study demonstrated that the rate of corrosion varied depending on the source of chlorides (continuous complete immersion as opposed to capillary suction from a small volume). However, the authors did not provide the results of the calibration. Additionally, the film deposited on the surface of the sensor partially deteriorated because of corrosion. In the described studies, the sensors were fixed around reinforcement bars. Chen and Dong [111], although they did not specify the type of sensors they used, described the operation of the devices using the ANSYS software and proposed a conversion coefficient between wavelength and deformation. They pointed out that the coefficient depends on the thickness of the concrete cover, and is close to 0.829, when the concrete thickness is five times higher than the diameter of the reinforcing bars. Gao et al. [112] fixed the sensors perpendicular to the axes of the reinforcing bars. As a result, using the gravimetric weight loss method, they obtained the relationship between reflected wavelength change from the grating and the weight loss rate of rebars caused by the formation of corrosion products. The authors also determined the time of corrosion initiation (when the readings of the sensors did not change) and the time of corrosion development (the signal changed monotonously over time).

Another important problem is the protection of embeddable sensors from mechanical damage occurring during the construction works and from the weight pressure of concrete [113]. Almubaied et al. [114] suggested putting an expanded polystyrene liner between the concrete and reinforcing bars. Although it helped to protect the sensor, the authors did not consider the reduction in adhesion between concrete and reinforcement and the effect of this factor on the load bearing capability of the whole structure. Jaafar et al. [115] used sensors by Photronix Technologies (M) Sdn. Bhd., with different types of concrete with a silicone gel protective coating. The authors determined the relationship between the Bragg wavelength and the changes in HCP, which was used for comparison. The suggested method ensured that the sensors functioned for at least a year under accelerated corrosion. Li et al. [116] tested FBG sensors with an epoxy protective layer and temperature sensor together with an acoustic emission sensor (AE, Micro-II Digital AE System, Physical Acoustic Corp, West Windsor Township, NJ, USA). The propagation of cracks in concrete was captured through a corresponding acoustic emission, and the surface strain was monitored by registering the increase on the amount of corrosion products using the FBG method. The study showed good agreement between the measurement results demonstrating that the method is promising for RC corrosion monitoring. By the end of the experiment, the sensors remained intact. We can thus assume that any protective coating resistant to the alkaline environment of concrete is able to protect FBG sensors from corrosion and destruction.

Luo et al. [106] demonstrated that the sensors used could provide information about the initiation of the corrosion process only when the amount of corrosion products increased 3–4 times as compared to the initial state. In other words, it is only possible to estimate the degree of corrosion, when the process is in progress. It is impossible to register the transition moment between the passive state and corrosion initiation, and therefore, it is impossible to take preventive measures to restore the passivity. We should also note that FBG sensors have a limited range within structures. A large number of sensors are required to monitor large structures. At the moment, such sensors are rather expensive to produce. If FBG sensors are to be widely used for monitoring of RC structures, their construction and the type of material used should be optimised in order to reduce their cost.

#### 3.1.2. Long Period Fibre Grating (LPFG) Refractive Index Sensors

The functioning of LPFG sensors is based on the modulation of the core refractive index resulting in attenuation bands on the receiver. Resonant wavelength changes depending on the reflection value, and reflects the corrosion activity of the environment. In their review, James et al. [117] described a promising idea of integrated fibre optic sensors which can be used for the parallel independent monitoring of several observables (temperature, bending, deformation, etc.). Let us consider several examples below.

Huang et al. [118] suggested a sensor coated with a thin layer of polyurethane and nanoscale iron/silica particles on single-mode optical fibres Corning SMFG28e. Quick laboratory tests allowed the authors to determine the correspondence between the accumulation of corrosion products and an increase in the resonant wavelength resulting from the corrosion of iron particles on the sensor. In other words, the authors used the nanoparticle coating as a sacrificial coating. The prediction error was 26%, which is a very good result for the localised corrosion of reinforcement bars in concrete. The polyurethane- coated sensor was used together with a sensor without polyurethane coating to control the temperature.

In study [119], Chen et al. suggested a sensor with a double coating (with an inner layer based on silver and an outer layer based on Fe-C), with the thickness of the layers varying from a few to several dozen μm. The laboratory experiments with a chloride aqueous solution demonstrated a linear dependence between the changes in the resonant wavelength and the weight loss of the outer Fe-C layer in certain ranges. In their next study [120], the authors used the suggested sensors to examine steel bars in mortar and obtained promising results for the early diagnostics of corrosion. Further studies will focus on the agreement of the sensor measurements with the gravimetric measurements of corrosion rate. However, the short service life of the sensor (24 h in aqueous environment and 2 weeks in mortar) means that it cannot be used for the long-term monitoring of real structures.

Thus, despite quite good results demonstrating the correspondence between the corrosion rate and the changes in the parameters of LPFG sensor signals, they cannot be used in the systems for the long-term monitoring of RC structures until the problem of corrosion resistance of nanomaterial coatings is solved. Since the use of such coatings is mandatory, one of the solutions may be the use of additional anti-corrosive protective films or more corrosion-resistant nanomaterials [121]. Although the considered sensors are more mechanically stable than FBG sensors, they still cannot be used to identify the moment of corrosion initiation, since they only register the accumulation of corrosion products on the surface of reinforcement bars.

#### 3.1.3. Brillouin Optical Time Domain Reflectometry Sensors

The distributed temperature and deformation can be measured by determining the dependence of the intensity of probe light on time and the distribution of the Brillouin frequency shift along the optical fibre.

Lv et al. [122] used Brillouin optical fibre time domain analysis (BOTDA) Ditest SAT 200 sensors to monitor the expansion strain from steel corrosion. The authors tried a new way to install the sensor: instead of placing it on the surface of the steel, they fixed it on a 5 mm thick layer of mortar covering the reinforcing bar. The whole construction was then covered with an additional layer of mortar. The sensor appears to be durable, highly sensitive, and has a wide measurement range. As a result of the study, the authors developed a damage coefficient for qualitative assessment from the early stages of the spread of corrosion products to the cracking of the inner layers of concrete. The obtained results can facilitate the further development of systems for monitoring RC structures. In order to use such sensors on real structures, it is necessary to develop a convenient method of sensor installation (the sensor should wrap around a reinforcing bar) and application of the interfacial layer of concrete.

Jagtap and Nayak [123] studied three different kinds of BOTDA sensors. The sensors differed in the way the fibre was fixed on the surface of reinforcement bars and the methods used to protect the sensors from the metal corrosion products (with a porous material layer serving as protection). The authors came to the conclusion that the described sensors can be used for long-term measurements. However, they did not specify which kind of sensors are the most optimal.

Fan et al. [124,125] suggested a distributed fibre optic sensor deployed in a helix pattern on a steel bar. The authors obtained dependencies which can be used to calculate the cross-section loss of reinforcement bars. The need to deploy the sensor along the whole surface of steel bars and the high possibility of deformation or destruction on a construction site are the main problems preventing its in situ application.

Scott et al. [126] suggested low-cost sensors that can be used to perform measurements, either on the surface of RC structures or within the concrete. The authors used two sensors simultaneously (one sensor for the deformation monitoring, and the other for the temperature monitoring). Both sensors were protected by a layer of polymer adhesive. The sensors were successfully tested in a laboratory environment.

Seo [127] described an experiment with BOTDA sensors used to monitor the strain and temperature of pile foundations on site. Monsberg et al. [128] reported comprehensive work on the design and implementation of a monitoring system where the fibre was installed in the above mentioned piles situated in a fault zone of the Semmering Base railway Tunnel (Austria).

The above considered BOTDA sensors are some of the first devices based on physical methods and are applied for the remote monitoring of steel reinforcement corrosion of real structures. They are quite popular because they allow for complete spatially distributed monitoring and sampling at distances of less than 1 mm [129]. However, the technology for winding such sensors around reinforcing bars, the serious influence of the temperature factor, and the high possibility of destruction during cracking of concrete limit the use of such sensors [106].

Shape Memory Alloys (SMA) Sensors are an increasingly popular subject for considerations and developments. Martensitic transformations triggered by temperature and stress occur in these metallic alloys. SMA are a class of alloys which can memorize their original shape. When the alloy is deformed, it can return to its original shape under the effect of temperature as a stimulus [130]. The pre-strained SMA is then embedded in a matrix in fresh concrete. After hardening of the matrix, the SMA is heated through resistive heating to activate. With an increase in tensile deformation, the electrical resistance of the alloy increases linearly, which makes it possible to estimate the crack width [131,132].

### 3.2. Elastic Wave Sensors

The relative magnetic permeability of steel is significantly (by over 100 times) higher than that of other components of RC, including the products of corrosion of reinforcing steel. By monitoring this difference, it is possible to identify the initiation and development of the corrosion process [133]. This is the effect that the functioning of elastic wave sensors is based on.

Xie et al. [134] tested surface acoustic wave (SAW) sensors. The sensor consisted of two printed circuit boards with reflecting meshes and an interdigital transducer (both made of gold) covered with a protective coating to prevent short circuits (in case the film of corrosion products grows). The sensor was embedded within concrete in close proximity to the reinforcement bars. The authors presented the results of quantitative assessment of the corrosion rate. However, they did not perform any calibration with regard to the actual weight and cross-section loss of the reinforcement. The construction of the sensor allows it to function without additional power sources and transfer data via a wireless channel. In order to use such sensors on site, it will probably be necessary to optimise their construction to make it easier to deploy them on reinforcing bars. It would also be reasonable to investigate other materials (less expensive than gold) for the mesh.

Sharma et al. [135] analysed the combined use of acoustic emission (AE) sensors and the ultrasonic guided waves (UGW) technique. The UGW sensors were deployed on reinforcement bars during the production of RC. These sensors proved to be more effective during the early stages of corrosion, when there are no cracks in concrete and the AE method does not work. During the formation of microcracks and their further growth, the AE method proved to be highly accurate in determining the regions with inner defects prior to the moment when they emerge on the surface. The authors only deployed the AE sensors for the duration of the study. The results of the laboratory experiments demonstrated the further prospects for this method. The laboratory experiments performed by Amiri et al. [136] demonstrated good agreement between UGW and HCP methods.

Although this above mentioned method looks promising, it is extremely sensitive to noise from external sources. It is also difficult to determine the relationship between the degree of corrosion and the level of signal, especially during the early stages of corrosion, when the defects in concrete are not yet visible [106,137]. A number of studies have been carried out to solve these problems [138,139,140], but the ultimate solution remains to be found. These problems must be solved before such sensors are used in real-life engineering structures. It is also necessary to determine the most optimal way to deploy the sensors, interpret data from large constructions, etc.

Du et al. [141] analysed all-optical photoacoustic sensors converting light energy into ultrasound waves. The sensors are based on nanocomposites of gold and a multimode optical fibre. The sensors were mounted on the surface of reinforcement bars. The sensitivity of the suggested system allowed the authors to determine corrosion loss starting from 0.02 g.

#### Piezoelectric Sensors

Ultrasonic methods and embeddable piezoelectric sensors are becoming a popular solution for the monitoring of corrosion of reinforcement steel in RC structures. Piezoelectric sensors generate acoustic vibrations induced by electric voltage and electric charges induced by acoustic waves.

Peng et al. [142] suggested a piezoelectric sensor based on ceramics (lead zirconate titanate) and composite materials used to isolate the sensor, screen noise, and enhance the sensor’s signal. The authors assessed the corrosion condition and the degree of damage to steel reinforcement based on the signal’s amplitude. The authors believe that the main drawback of this method is the unidirectionality of the output and input, which makes it difficult to interpret the data in real-life corrosive environments with reinforcement bars arranged in various directions. In their further studies, the authors plan to focus on this problem.

Peng et al. [143,144] also analysed sensors based on lead zirconate titanate and modified Fe, Mn, Ca (PZT-4-type), and La, Nb (PZT-5-type). On the whole, their results are close to those obtained in [142].

Su et al. [145] suggested a self-powered wireless sensor network for the automated prediction of steel reinforcement corrosion. The authors used self-powered wireless piezoelectric sensors. They predicted the corrosion rate based on the modelling and monitoring of corrosion data for five years.

Sriramadasu et al. [146] used piezoelectric wafer transducers based on ceramics by PI Ceramic (PIC 151) with ultrasonic guided waves. The study assessed the signal characteristics of the longitudinal and flexural-guided wave modes of the sensors attached to the surface of steel reinforcement bars. As a result, the authors established the stages of the initiation and development of corrosion. Quantitative characteristics of the destruction process of metal will be the focus of further studies.

Kaur and Bhalla [147] investigated piezoelectric sensors based on AD5933 converters embedded within concrete. The suggested set-up provides information about the strain within concrete, and harvest enough energy to support the conversion and transfer of data without using external power sources. Kocherla et al. [148] demonstrated that this method can be used to identify crack openings smaller than 10 μm.

Chen et al. [149] studied commercially available sensors Model No. GU14095A0-25TR-L42 (Shenzhen Yinghai, Ltd., Shenzhen, China). The authors believe that piezoelectric sensors are more promising than ultrasonic sensors due to their lower cost.

Xu and Tang [150] used sensors based on PZT 5 ceramic. The authors obtained good agreement between the relative amplitude of the measured signal and the corrosion rate, when the rebar cross-section loss is from 0 to 7%. With a more serious destruction level, the agreement is not so good, because the adhesion between the reinforcement bars and concrete decreases due to the accumulation of corrosion products.

Acoustic sensors are generally more sensitive than FOS during the early stages of corrosion, when the amount of corrosion products is still not very large. However, the use of such sensors in real engineering structures is problematic due to their high sensitivity to noise. Further studies may develop effective methods to eliminate noise and present new commercialised solutions.

### 3.3. Hall Effect Sensors in an Electromagnetic Field

The magnetic permeability of carbon steel is 100 times higher than that of other components, i.e., concrete, water, air, and iron oxide. It is possible to estimate the formation of corrosion products on the surface of reinforcing bars by measuring the magnetic properties of the system. The most common sensors used for such measurements are Hall effect sensors.

Zhang et al. [133] used a Hall effect sensor (SS495A) in an electromagnetic field (EMMA). The authors fixed three Hall effect sensors at a specific distance from the reinforcement bar and immersed the set-up in concrete. The first sensor was fixed closer to the reinforcing bar with the poles of the electromagnet located at the edges of the bar. HCP and AE methods were used for comparison. The authors obtained a linear dependence between the weight loss of the metal and the voltage increment detected by the Hall effect sensor due to the variation of magnetic induction.

Li et al. [151] developed an EMMA system with 24 Hall effect sensors and a U-shaped electromagnet around the examined construction. The system was used to detect the formation of corrosion products on the surface of steel reinforcement. The results corresponded well with the results of EIS. However, the accuracy of detection of uniform corrosion was higher than the accuracy of detection of localised corrosion. The suggested U-shaped electromagnet can only be applied on site to columns and bars which allow arranging three sources of electromagnetic field around them. Otherwise, it will be necessary to modify the system or apply other monitoring techniques.

The authors also analysed combined use of electromagnetic sensors and acoustic emission apparatus [152]. The AE sensors were used to detect cracks in concrete induced by the corrosion products. The study demonstrated that there was good agreement between the measurement results and the actual condition of the examined structures. In their latest study [153], the authors focused on combined application of electromagnetic sensors and digital image correlation technique. A combination of physical methods and computer processed images of the condition of the surface can enhance the diagnostic abilities of the system. We should also note that the authors elaborated the design of their sensors, which were, at first, too large. At the moment, they are presented as compact circuit boards and can be commercialised. The digital image correlation technique can be used to examine an arbitrary region selected by the operator, while the EMMA system only monitors the regions where the sensors are located.

Considering the aforementioned, the digital image correlation technique is a more technologically advanced method of monitoring. Provided that the cameras recording images and videos are deployed correctly, the monitoring can be carried out remotely. Similar results are obtained when using the thermal image data technique described by Na et al. [154], Kobayashi and Banthia [155], and Kato [156]. Omar and Nehdi [157] used a thermal imaging system born by an unmanned aerial vehicle to monitor bridges.

We should note, however, that such techniques are associated with specific problems that have to be solved. Namely, the effect of the electromagnetic field on the state of the reinforcement in concrete and the issue of monitoring large structures, which cannot be performed by using a portable electromagnet.

The above considered physical methods and the sensors based on them allow for the accurate assessment of the accumulation of corrosion products on the surface of steel reinforcement bars in concrete during the stage of corrosion development. However, they are less sensitive during the stage of corrosion initiation when the rust film is yet small. Considering this, the most promising solutions appear to be integrated monitoring systems, which combine electrochemical sensors (used to detect the corrosion initiation moment) and physical methods (used to analyse and predict the development of corrosion, the beginning of cracking, flaking of the concrete cover, or collapse of the whole structure).

## 4. Integrated Sensor Systems

The durability of RC depends on a number of factors. Therefore, the monitoring systems should combine several sensors measuring different physicochemical parameters in real time [158]. The best systems for monitoring RC structures are integrated modular systems [159]. The theoretical basis for such systems is a generalised model of corrosion for steel in concrete which comprises all the stages of the process.

**Figure 4:**
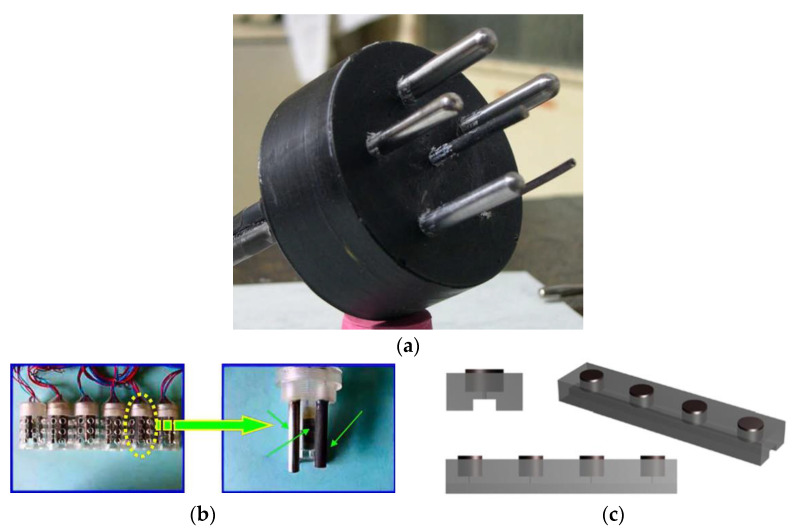
Examples of multifunctional sensors: (**a**) Duffó and Farina [160]; (**b**) Qiao et al. [164]; (**c**) Jeong and Kim [166].

Duffó and Farina [160] suggested multifunctional sensors, which can be used to monitor the HCP, the corrosion rate (using the LPR methods), the electrical resistivity of concrete, the concentration of chlorides, and the temperature (Figure 4a). The authors designed special software which detects the moment of transition from a passive state to corrosion. The authors stressed that the system is low-cost and can be applied to both new and rehabilitated structures. The system was tested in situ on a large model structure. Titanium rods from a standard cathodic protection system were used as the reference electrode. The surfaces of the rods were modified with iridium and tantalum.

Martínez and Andrade [161] experimented with sensors that had similar functions. The sensors were embedded in concrete cubes and in a real bridge. The reference electrodes were systems based on Ti, MnO_2_, Ag, and Pb.

Lu and Ba [162] performed laboratory testing of a multi-sensor system, which can monitor the HCP, the macrocell current, the concentration of chlorides, the electrical resistivity of concrete, and the rate of corrosion (with the LPR method) at the same time.

Yu and Caseres [163] developed a multi-electrode system that can be used to monitor the chloride concentration, the HCP, pH, the electrical resistivity of concrete (with a Wenner probe), and the rate of corrosion (with the LPR method). The control samples were used to monitor the weight loss. Laboratory testing was performed on concrete specimens.

Qiao et al. [164] used five-electrode sensors with two graphite electrodes (a reference electrode and an auxiliary electrode) and three working electrodes made of low-carbon steel. They were used to estimate HCP, GPT, and electrochemical noise (Figure 4b). The authors also designed software to process the noise signal. The results are in good agreement.

There are also examples of effective combinations of electrochemical and physical methods. Thus, Arndt et al. [165] used microcells in an anode-ladder sensor, ground penetrating radar, and active inductive thermography to detect the corrosion of steel reinforcement bars in concrete specimens. The HCP technique was used for comparison. The obtained results were combined and assessed together, which allowed for a more accurate evaluation.

Jeong and Kim [166] tested a four-electrode sensor for the simultaneous monitoring of HCP, CR, and current density by means of the LPR method (Figure 4c). A platinized titanium electrode was used as the reference electrode.

Ramón et al. [167] suggested an Integrated Network of Sensors for Smart Corrosion Monitoring (INESSCOM) and tested it on a model. The system is comprised of a standard three-electrode cell (with several working electrodes), and a new mode of signal transmission which allows for simultaneous measurement of the electrical resistivity of concrete and the corrosion current density.

The present study does not consider a number of methods, for instance, harmonic analysis and electrochemical noise, because there are no monitoring sensors based on them. The list of monitoring systems presented here may be incomplete. At the moment, we continue to collect and analyse information about the current state of research in the field.

## 5. Conclusions

Non-destructive electrochemical methods for assessing the health of steel reinforcement bars in concrete have been studied for 50 years. As a result, a number of standards and regulations have appeared, as well as a series of commercially available systems for in situ monitoring. This is why sensors based on electrochemical methods are more often used to monitor real-life structures.

Quality electrochemical methods (HCP, CR) allow to quickly and fairly accurately determine areas with a high probability of corrosion of the reinforcement. This is convenient for inspections with the participation of an inspector, but it gives less valuable information for sensors implanted in concrete, because information about the rate of destruction of reinforcement is more valuable. The electrochemical LPR method is the most common and allows to estimate the corrosion rate of steel reinforcement. This is reflected in a significant number of sensors and monitoring systems. Other quantitative electrochemical methods (GPT, EIS) are less common due to more complex equipment and complex integration into data analysis.

Non-destructive physical examination methods for the detection of corrosion in steel reinforcement bars have been studied in detail for the last 10–15 years. The principle of operation of sensors based on physical methods is often based on more indirect measurements than for electrochemical ones. The most common physical fibre optic sensors demonstrate good results at the stage of corrosion development, when adhesion at the reinforcement/concrete boundary deteriorates and cracks of various opening widths form. A large number of design options and applied principles (FBG, LPFG, BOTDA, SMA) give a good prospect for the practical implementation of these devices in the form of ready-made commercial solutions. The situation is similar with sensors based on elastic waves and the Hall effect. The studies should result in a number of standards regulating the principles of application of the most effective approaches.

The most effective monitoring systems combine electrochemical and physical methods and sensors based on them. The choice of a particular combination is based on the complexity and importance of the structure, the adversity of the operating conditions, and the economic feasibility of the methods.

## Figures and Tables

**Table 1:** Range of HCPs of steel reinforcements in concrete used for the assessment of the corrosion condition (with regard to the copper/copper sulphate reference electrode at 20 °C).

Range of Values *E*_cor_, mV	Corrosion Condition of the Reinforcement
>−200	Passivity with a probability of 90%
−200…−350	Undefined state
<−350	Corrosion with a probability of 90%

**Table 2:** Range of CR values used to estimate the probability of corrosion of steel reinforcement bars in concrete [34].

Range of Values CR, Ωm	Risk of Corrosion of Reinforcement (for 20 °C)
<100	high
100…500	moderate
500…1000	low
>1000	negligible

**Table 3:** Values of corrosion current density (*i*_cor_) and cross-section loss (Δ*l*) of reinforcement for assessing the condition.

Range of Values		Corrosion Rate
*i*_cor_, μA·cm^−2^	Δ*l*, μm·y^−1^	
≤0.1	≤1.16	passive state
0.1…0.5	1.16…5.80	low
0.5…1.0	5.8…11.6	moderate
>1.0	>11.6	high
